# Identification of Xylem Occlusions Occurring in Cut *Clematis* (*Clematis* L., fam. *Ranunculaceae* Juss.) Stems during Their Vase Life

**DOI:** 10.1100/2012/749281

**Published:** 2012-07-31

**Authors:** Agata Jedrzejuk, Julia Rochala, Jacek Zakrzewski, Julita Rabiza-Świder

**Affiliations:** ^1^Department of Ornamental Plants, Faculty of Horticulture and Landscape Architecture, Warsaw University of Life Sciences, Nowoursynowska 166, 02-787 Warsaw, Poland; ^2^Department of Forest Botany, Faculty of Forestry, Warsaw University of Life Sciences, Nowoursynowska 166, 02-787 Warsaw, Poland

## Abstract

During the vase life of cut stems obstruction of xylem vessels occurs due to microbial growth, formation of tyloses, deposition of materials in the lumen of xylem vessels and the presence of air emboli in the vascular system. Such obstructions may restrict water uptake and its transport towards upwards thus lowering their ornamental value and longevity of cut flowers. *Clematis* is a very attractive plant material which may be used as cut flower in floral compositions. Nothing is known about the histochemical or cytological nature of xylem blockages occurring in cut stems of this plant. This study shows that in *clematis*, tyloses are the main source of occlusions, although bacteria and some amorphic substances may also appear inside the vessels. A preservative composed of 200 mg dm^−3^ 8-HQC (8-hydroxyquinolin citrate) and 2% sucrose arrested bacterial development and the growth of tyloses. This information can be helpful in the development of new treatments to improve keeping qualities of cut *clematis* stems.

## 1. Introduction

*Clematis* is used in Europe mostly as a climber plant, but, because of its beautiful flowers, this genus may also provide cut flowers for floral compositions. It is used as such in the United States; the European flower market still lacks suitable *clematis* cultivars and methods allowing to control thier postharvest quality. This creates a broad opportunity for the European growers and breeders of ornamental plants. Several Polish cultivars are proving themselves to be potential sources of a good cut ornamental material. 

The postharvest life of *clematis* ranges between 2 and 14 days, and it depends on a cultivar. The standard preservative to effectively prolong the vase life of *clematis* is a solution of 200 mg dm^−3^ 8-hydroxyquinolin citrate (8HQC) with 2% sucrose [1], but more advanced studies are needed to develop preservatives and treatments suitable for *clematis* during all steps of the market chain. Proper water balance in cut stems is crucial for the flower postharvest longevity, and blockages occurring in vessels disturb it by limiting water uptake and transport to the flower. The main cause of reduced water uptake in cut stems is obstruction of xylem vessels by microbial growth, formation of tyloses, deposition of materials in the lumen of xylem vessels, and the presence of air emboli in the vascular system [2, 3]. 

The invasion of the dead lumens of tracheary elements by living parenchyma cells (formation of tyloses) is a well-known response to infection by pathogens and to wounding [4]. It is often accompanied or followed by the transformation of gums and tannins which add to the strength and durability of the composite polymers. The nature of such material was investigated cytologically, revealing the presence of pectic elements, callose, or lignin-like molecules [5–9]. Such material is produced by the plant in response to invasion by the bacteria [10–14] or in response to phytotoxins produced by bacteria [15]. 

This study was conducted to provide cytochemical and immunohistochemical information on vessel occlusions and the involvement of tyloses, gels, or gums in their formation in cut *clematis* stems kept in different vase solutions.

## 2. Material and Methods

### 2.1. Plant Material

The study was done on flowering stems of *clematis* (*Clematis* L.) kindly provided by Mr. Szczepan Marczyński and Władysław Piotrowski from the plant nursery in Duchnice near Warsaw. The choice of the cultivar was based on observations of the vase life length made by Skutnik and Rabiza-Świder [1, 16] and previous anatomical observations of stems of five different cultivars, of which two were short-lasting (“Andromeda” and “Viola”), one medium lasting (“Isago”), and two long-lasting (“Solidarność” and “Silver moon”). Additionally, anatomical studies of stem blockage formation were done in all five cultivars, while the histochemical, immunohistochemical and cytological identification of the nature of blockages was done in only one, cv. “Solidarność.”

Flowering stems were harvested at the same stage of development, immediately transferred to laboratory and trimmed to 20 cm. Shoots were placed in distilled water or the standard preservative composed of the bactericide 8HQC + sucrose (SUC) which was tested as the most effective preservative [1, 16]. There were eight shoots in each treatment, individually tagged and treated as individual replications. The experiments were conducted at 18–20°C and a 12 h photoperiod, provided by luminescence light with a quantum irradiance of 25 mol m^−2^ s^−1^. The relative air humidity was maintained at 60%.

### 2.2. Anatomy, Histochemistry, and Immunolocalization

Stem ends *ca* 5 mm long were sampled on three dates: just after harvest (control, day 0), after 7 days (wilting of flowers kept in distilled water, term I), and after 12 days, when wilting and loss of a decorative value occurred in flowers placed into the preservative (term II). On terms I and II, the stem fragments were collected from both treatments (distilled water, preservative).

The specimens were fixed in the PFA fixative: 4% paraformaldehyde (Sigma), 0.4% DMSO (Sigma), 0.05M phosphate-buffered saline (PBS) (pH 7.0), DEPC-treated water (Sigma) for 12 h under 0.6 atm. Fixed samples were washed twice for 30 min. each in the phosphate-buffered saline (PBS), dehydrated in the graded ethanol series (30%, 50%, 70%, 80%, 95%, 100%), each series for 1 h in RT (room temperature), and twice in Histoclear (Histochoice Clearing Agent, Sigma) for 30 min each. Paraplast pellets (Sigma) were added to the last series of Histoclear in the paraffin oven, twice a day for 5–7 days, in temperature 56–58°C, until the Histoclear evaporated completely. In the last step, specimens were embedded in clear Paraplast (Sigma). Semi-thin sections (10 *μ*m) were sectioned on a rotary microtome (Reichert Jung). All preparations were made on the RNase, DNase-free objective slides (Thermo Scientific MenzelGläser, Superfrost Plus), and dried at 42°C for 2–4 days. 

For general anatomical identification of xylem occlusions in *clematis* stems, permanent slides were stained using the safranin—fast green method. For the histochemical identification of xylem occlusions, slides were stained as listed in Table 1.

For some cell wall components, sections were incubated with monoclonal primary antibodies Jim 5, Jim 7 (detection of homogalacturonans), Jim 11, Jim 12, Jim 20 (detection of extensins) synthesized by Dr Knox, Centre of Plant Sciences, University of Leeds, Leeds, UK (details at http://www.plantprobes.net/). Primary antibodies diluted 1 : 20 in PBS were applied for 2 h at 37°C. Secondary, antiRat IgG antibody labeled with the alkaline phosphatase (SIGMA) was applied for 2 h in 37°C, and slides were incubated with the nitroblue tetrazolium chloride and 5-bromo-4-chloro-3′-indolyphosphate p-toluidine salt (NBT/BCIP) (Sigma) diluted in 100 mM Tris, 100 mM NaCl, 50 mM MgCl_2_, for 2 h in the dark. All observations were made using olympus BX41 bright field microscope.

### 2.3. Electron Microscopy (EM)

For conventional EM observations, stem fragments were fixed for 6 h in 2.5% glutaraldehyde (Sigma) buffered with 0.1 M cacodylate buffer, pH 7.2, rinsed in the same buffer and postfixed for 2 h in 1% osmium tetroxide (Merck). Samples were dehydrated in a graded series of alcohol followed by dehydrated acetone and embedded in Epon (Fluka). After thin sectioning, samples were stained with 3% uranyl acetate and Reynold's lead citrate and examined under a JEOL JEM100C transmission electron microscope.

### 2.4. Statistical Analyses

The xylem vessel data were tested using analysis of variance (Anova 1) with the Stagraphics 4.1. program. Means were compared using the Duncan's multiple range test at *P* = 0.95.

## 3. Results and Discussion

### 3.1. Anatomical Organization of *Clematis* Stems

**Figure 1:**
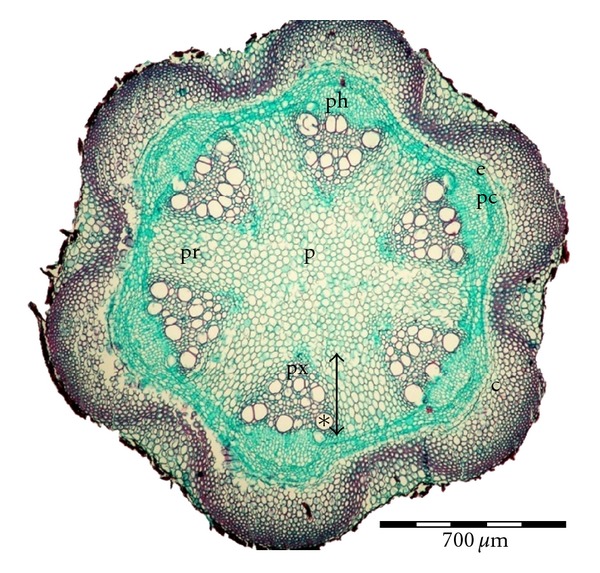
Transverse section of *Clematis* “Solidarność” stem, p: pith, pr: pith rays, ph: phloem, px: protoxylem, ∗: vessel of metaxylem, *↕*: xylem, e: endodermis, c: cortex with lamellar collenchymas, pc: pericycle.

Stems of different cultivars of *clematis* contain between six to twelve primary vascular bundles with the diameter in the metaxylem between 17.9 and 110.5 *μ*m (see Table 2). Stems of cv. “Solidarność” contain six primary vascular bundles with well visible cambium between the phloem and xylem zones (Figure 1). Cambium consists of 3-4 meristematic cells in a radial arrangement. At the inner side, the primary xylem contains small vessels of protoxylem and, on the outside, a rather wide region of metaxylem with large diameter vessels (Figure 1). The primary rays have parenchymatous cells with 3–5 layers in tangential direction. The pith region is wide and parenchymatic and occupies mostly the center part of the stem. At the outer side of the vascular bundle, there is a multicellular, parenchymatic pericycle. The cortex contains a parenchymatous endodermis, a layer of parenchyma cells, and a ring of lamellar collenchyma cells. The diameter of vessels in metaxylem ranges from 26.4 to 66.7 *μ*m. 

According to van Meeteren et al. [17], the vessel diameter may affect the duration of the postharvest life. This is based on the fact that wide vessels are more efficient in water transport [2, 18], and the xylem occlusions do not block the entire lumen of the vessel. Our preliminary research (Table 2) showed that diameter of primary xylem vessels seems to be associated with the length of postharvest life. Observations on all five cultivars showed a similar architecture of stem anatomy, except for the number of primary xylem vessels and their diameters. According to Skutnik and Rabiza-Świder [1, 17] the cut stems of cv. “Solidarność” are long-lasting and their wilting in distilled water occurs after 10 days. 

### 3.2. Cytological Identification of Xylem Occlusions

**Figure 2:**
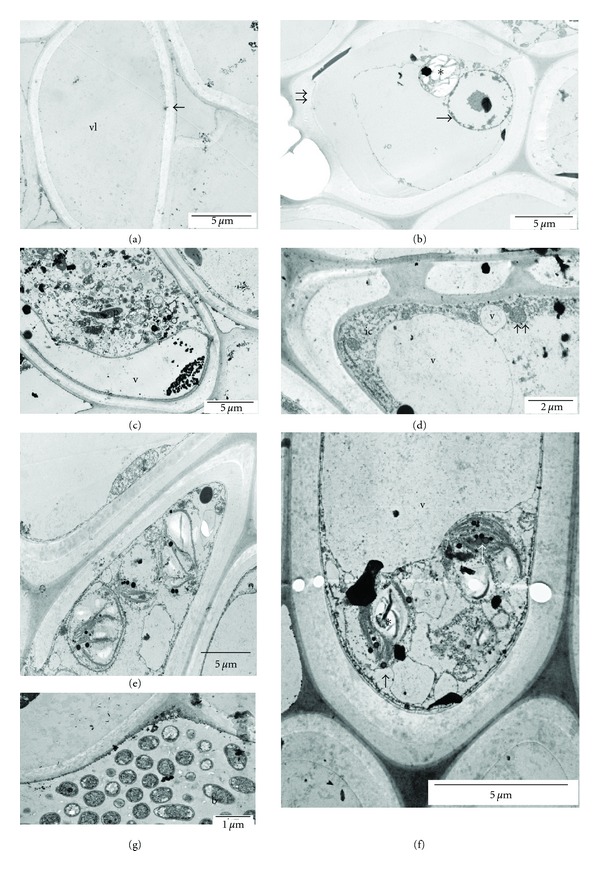
Ultrastructural identification of xylem occlusions in stems of *Clematis* “Solidarność” (a) empty vessel, control, (b) young tylose, stems kept in distilled water, date I, (c) mature tyloses, stems kept in distilled water, date II, (d) young tylose, stems kept in 8HQC with 2% sucrose, date I, (e) and (f) mature tylose, stems kept in 8HQC with 2% sucrose, date II, (g) bacteria in blocked vessels, stems kept in 8HQC with 2% sucrose, date II. Vl: vessel lumen, *←*: cell wall, ∗: amyloplast, →: degrading nuclei, *⇉*: tylose cell membrane, P: plastid, ic: intact cytoplasm, v: vacuole, *⇈*: mitochondria, ↑: lipid bodies, b: bacteria, Control: term 0, Term I: after 7 days of postharvest life (wilting of flowers kept in distilled water), Term II: and after 12 days, when wilting and loss of a decorative value occurred in flowers placed into the preservative.

In freshly harvested control stems, the xylem vessels were free of occlusions. The thickness of cell walls ranged from 1.0 to 1.2 *μ*m (Figure 2(a)). After seven days in distilled water (date I), xylem vessels were blocked primarily by tyloses and to a lesser extent by bacteria. Tyloses contained mostly amyloplasts, and ther nuclei showed well-advanced fragmentation (Figure 2(b)). On this collection date, no amorphic or jelly substances were observed in the vessel lumen, but, in 5–7% of the specimen examined, some bacteria were present. On the second collection date, 12 days of vase life in water, tyloses filled nearly the entire volume of xylem vessels. Tyloses contained all components of parenchymatic cell matrix, for example, plastids (Figure 2(c)), mitochondria, plastids, degenerated lipid bodies (data not shown), and plenty of amorphic substances probably originating from degenerating cytoplasm (data not shown). Bacteria were responsible for 27–30% of blocked vessels. In most cases, the vessels blocked by bacteria were free of tyloses.

After seven days of vase life, the stems kept in the preservative solution contained only several tyloses (5.6–7.8% of all observed specimen) with very thin membranes (0.03 *μ*m) and big vacuoles, filling most of the tyloses' spaces. Tyloses also contained mitochondria, autophagosomal vacuoles and intact cytoplasm near the cell wall (Figure 2(d)). Tyloses did not fill the entire lumen of xylem vessel. On the second collection date (after 12 days of vase life), the thickness of the cell membranes was *ca* 0.1 *μ*m. Tyloses contained big amyloplasts (Figure 2(e)) and traces of lipid bodies which preserved their shape and did not degenerate (Figure 2(f)) as they did in stems placed in distilled water (data not shown). Large vacuoles were also observed, similarly to flowers kept in water (Figure 2(f)). Apart from tyloses, some bacteria were present in 10–12% of blocked vessels as well as tubular material at the border of the xylem cell wall (Figure 2(g)). 

In both treatments, young tyloses (those from collection date I) usually were globular in shape and elongated during the tylose development. According to Clérivet et al. [15], globular-shaped tyloses are outgrowths of the vessel-associated parenchyma cells, which balloon through pit cavities into adjacent vessel elements. They are generally considered as a primary defense mechanism during vascular attacks and hamper the pathogen transportation within xylem vessels [18–21]. Our observations show that the main source of xylem blockage in cut *clematis* stems is tyloses and that their development is delayed when stems are kept in a standard preservative containing 8HQC + SUC. Even though some bacteria and amorphous material were present in observed specimens, their spread was reduced by the preservative by nearly one half relative to distilled water. The amount of amorphous material in the xylem lumen was localized only near the secondary walls of xylem vessels and was probably connected with the activity of bacteria and a degradation of cell components.

### 3.3. Anatomical, Histochemical, and Immunohistochemical Identification of Xylem Occlusions

**Figure 3:**
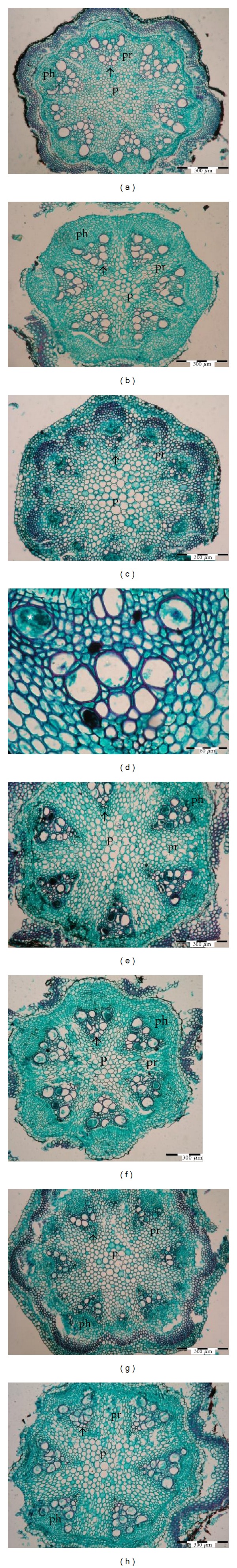
Identification of xylem occlusions in short-lasting (Andromeda) and long-lasting (Solidarność) cultivars of *Clematis* by staining with fast green and safranin. (a) and (b) Control stems with xylem vessels free of any occlusions in Andromeda (a) and Solidarność (b). (c) and (d) Stems kept in distilled water, term I with xylem vessels half and completely blocked by the occlusions in Andromeda (c) and Solidarność (d). (e) and (f) Stems kept in distilled water, term II with xylem vessels half and completely blocked by the occlusions in Andromeda (e) and Solidarność (f). (g) and (h) Stems kept in 8HQC + 2% sucrose, term II with xylem vessels half and completely blocked by the occlusions in Andromeda (g) and Solidarność (h). c: cortex with lamellar collenchyma, p: pith, pr: pith rays, ph: phloem, arrow: xylem vessel, ∗: blocked xylem lumen. The choice of the cultivar was based on observations of the vase life length made by Skutnik and Rabiza-Świder [1, 16] and previous anatomical observations of stems of 5 different cultivars. Two of them were characterized as short-lasting (“Andromeda”, “Viola”), one medium (“Isago”), and two long-lasting (“Solidarność” and “Silver moon”) cultivars. Control: term 0, term I: after 7 days of postharvest life (wilting of flowers kept in distilled water), term II: after 12 days, when wilting and loss of a decorative value occurred in flowers placed into the preservative.

In the freshly harvested stems (collection date 0), no traces of mechanical vessel blockage or gel occlusions were observed in any of the five cultivars studied (Figures 3(a) and 3(b)). After seven days of vase life (collection date I), in stems kept in water, completely blocked vessels presented *ca* 5.06%–7.88% and half- blocked vessels *ca* 8.64–11.36% of the total vessel number in all observed cultivars (Figures 3(c) and 3(d), Table 3) Blocked vessels were observed only in the metaxylem. After 12 days of the vase life in distilled water (collection date II), completely blocked vessels represented about 10–18.94% and the half-blocked vessels about 9.5–16.6% of the total vessel number (Figures 3(e) and 3(f), Table 4). Completely and half-blocked vessels were present both in the proto and metaxylem (Figures 3(e) and 3(f)). 

In stems kept in the standard preservative on the collection date I, completely blocked vessels were blocked in 5.7% in the short-lasting cv. “Andromeda” and in 3% in cv. “Isago.” The remaining three cultivars had less than 1% vessels blocked (Table 3); half blocked vessels were present in 6.2–33,8%, and they were seen in the proto and metaxylem (Table 3). On the collection date II, completely blocked and half-blocked vessels represented 3%–29% and 22.3–37.5% of the total number of vessels observed, respectively, and they were present both in the proto- and in metaxylem (Figures 3(g) and 3(h), Table 4). 

There was no correlation between the vessel diameter and proportion of blocked or half-blocked vessels. In stems stored in distilled water (collection date I), the short-lasting cv. “Andromeda” had the highest number of completely blocked vessels (7.8%), but cv. “Viola,” another short-lasting cultivar had the lowest number of completely blocked vessels (5.1%). For the collection date II, the highest number of completely blocked vessels was in the mid-lasting cv. “Isago,” around 19%, and the lowest number of completely blocked vessels was in the short-lasting cv. “Andromeda” and the long-lasting cultivar “Solidarność” (12.7%). Stems stored in 200 mg dm^−3^ 8HQC with 2% sucrose in sampling date II showed a clear effect of the preservative: the short-lasting cv. “Andromeda” had 28.3% of completely blocked vessels while the long-lasting cv. “Silver Moon” had 2.9% of such completely blocked vessels. Skutnik and Rabiza-Świder [1, 17] rate cv. “Andromeda” and “Viola” as short-lasting, and both had the lowest diameters of the metaxylem vessels, 17–54 *μ*m. Long-lasting cultivars had larger diameters of xylem vessels, 27–110 *μ*m. Their better postharvest longevity may be associated with a better water hydraulic conductivity through wider lumen of the vessels. We have observed that on sampling date II, the number of completely blocked vessels was higher in stems kept in 8HQC + SUC than in stems kept in distilled water in only one, short-lasting cv. “Andromeda” (Table 4). In cv. “Viola” and “Isago,” the number of completely blocked vessels in the stems kept in 8HQC + SUC was lower when compared with stems kept in distilled water and it was significantly lower in the long-lasting cv. “Solidarność” and “Silver Moon” (Table 4). 8HQC + SUC significantly reduced the numbers of completely blocked vessels for the sampling date in all studied cultivars, when flowers were still decorative and at the onset of wilting (Table 3). The number of half-blocked vessels in the stems treated by 8HQC + SUC was higher than in stems stored in distilled water in all observed cultivars in sampling dates I and II (Tables 3 and 4). This suggests that 8HQC + SUC delays the development of tyloses compared to distilled water but does not stop it completely. 

### 3.4. Histological and Immunohistological Identification of**** Xylem Occlusions in cv. “Solidarność”

#### 3.4.1. Polysaccharides

**Figure 4:**
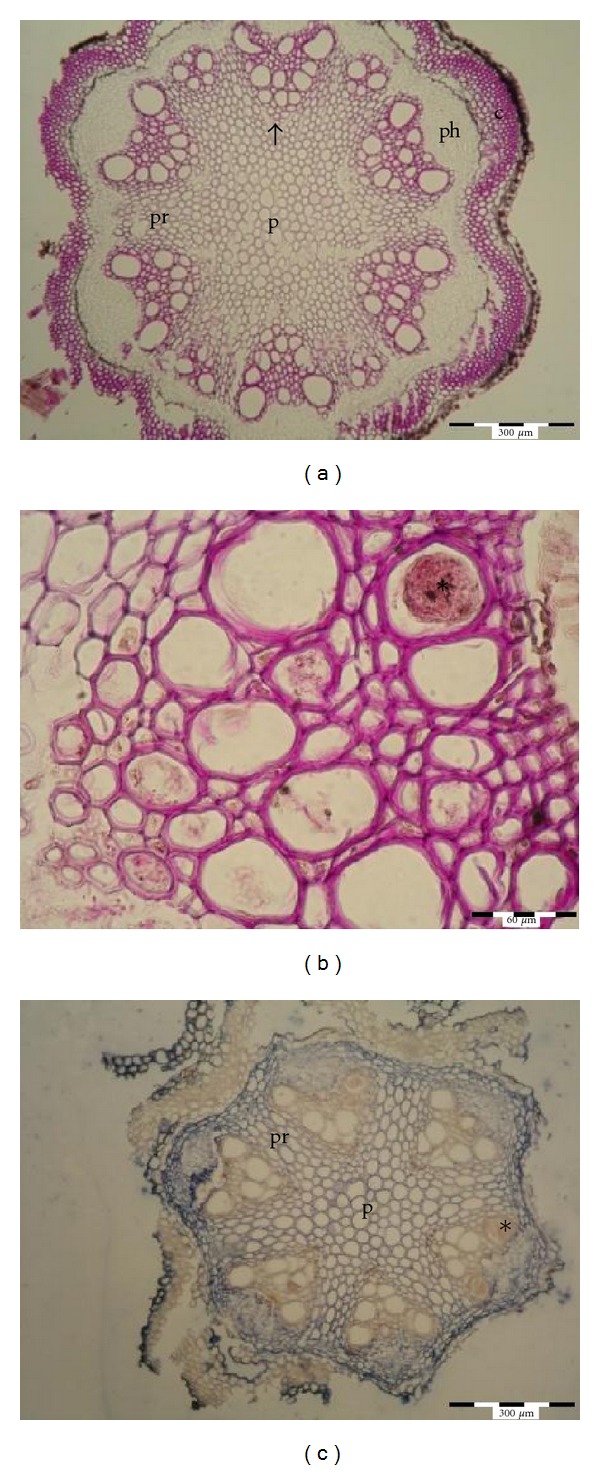
Histochemical identification of polysaccharide components and homogalacturonan epitopes in xylem occlusions in stems of *Clematis* “Solidarność” by using PAS reaction and Jim5 antibody. (a) and (b) Identification of polysaccharide components in xylem of control stems (a), stems kept in distilled water (b), (c) identification of homogalacturonan epitopes in xylem of stems kept in distilled water. C: cortex with lamellar collenchyma, p: pith, pr: pith rays, ph: phloem, arrow: xylem vessel, ∗: blocked xylem lumen.

In controls (sampling date 0) free of any xylem pollutants, polysaccharides were detected in cortex, proto- and metaxylem cell walls with parenchymatic cells of the primary rays. The red color of the PAS reaction was also visible in pith rays, but the color intensity suggested lower accumulation of polysaccharides in pith cells. No color reaction was observed in the phloem and cambium. The epidermis stained brown which is the natural color of the tissue and not the test reaction (Figure 4(a)). 

In distilled water after 7 days of vase life (collection date I), the PAS reaction produced clear, red color of blocked vessels. The color intensity ranged from light in the half-blocked vessels to intense in the completely blocked vessels (Figure 4(b)). On the second collection date (after 12 days), strongly red stained vessels were present also in protoxylem (data not shown).

On both collection dates (after 7 and 12 days), in stems kept in the standard preservative, only light red color was visible in the blocked and half-blocked vessels. In this treatment, only metaxylem showed weak color coming from the reaction (data not shown). 

Homogalacturonan epitopes were recognized by JIM 5 and JIM 7 antibodies [22]. Histological immunolocalization of homogalacturonans showed strong, blue coloration in the phloem, cambium, pith, and particular cells of primary rays when specimens were incubated with the Jim 5 antibody. No evidence of homogalacturonans was present in xylem occlusions observed in stems placed either in distilled water (Figure 4(c)) or in 8HQC + SUC on any collection date. The Jim 7 antibody gave a much weaker signal than Jim 5 antibody (data not shown).

Strong red color of the xylem occlusions after the PAS reaction in the stems kept in distilled water confirmed a high concentration of polysaccharides in the tyloses. Histoimmunochemistry did not localize any homogalacturonans in tyloses nor in the tubular material blocking xylem vessels. According to the literature [8, 15, 23], pectins concentrate in gels which may occlude xylem vessels, but their presence in tyloses is rather rare. In our study, no pectins were detected on the histochemical level, neither in tyloses nor in amorphous, extracellular material occluding xylem vessels. However, in some plants, tylose differentiation may correlate with the accumulation of pectins in parenchymatic cells of pith [15, 24]. In the stems of *clematis*, accumulation of pectin epitopes in pith rays was quite evident, but they were not observed in tyloses. 

#### 3.4.2. Proteins

**Figure 5:**
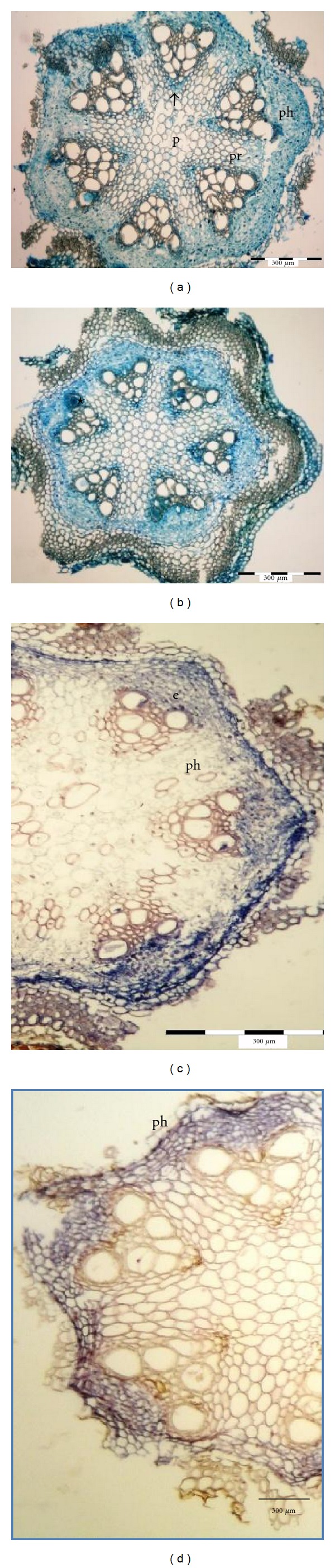
Histochemical identification of protein components and extensins in xylem occlusions in stems of *Clematis* “Solidarność” by using Bradford reagent and Jim11 antibody. (a) and (b) Identification of protein components in xylem of control stems (a), stems kept in distilled water (b), (c) and (d) identification of extensins in xylem of stems kept in distilled water (c) and 8HQC + 2% sucrose (d). p: pith, pr: pith rays, ph: phloem, arrow: xylem vessel, ∗: blocked xylem lumen, e: endodermis.

The presence of proteins in xylem occlusions was checked using the Bradford reagent. In control stems, an intensive blue color was visible in phloem, cambium, and the parenchymatic cells surrounding the primary xylem. No evidence of proteins was observed in pith rays, xylem vessels, and collenchyma (Figure 5(a)). On both collection dates during the vase life, in stems kept in distilled water, proteins were visible in all blocked vessels (the half- and completely blocked) both in the proto- and metaxylem (Figure 5(b)). 

In stems kept in the standard preservative blocked vessels colored light blue on the sampling date I and strongly blue on the sampling date II. The coloration intensity was equally strong in the completely and half-blocked vessels. Proteins are very often present in the gels or gums occluded into the lumen of the vessel. Tyloses mostly include polyphenols with their serious antiseptic properties. The tyloses observed here also included proteins probably transported as an extracellular material from the parenchymatic cells surrounding the primary xylem.

Extensins were identified with the extensin-specific antibodies (Jim 11, Jim 12, Jim 20). None of the antibodies bound to the xylem occlusions. When Jim11 was used, the dark blue color indicating the presence of extensin was not detected in the phloem and endodermis (Figures 5(c) and 5(d)). Jim 12 and Jim 20 did not detect any extensins at the histological level. The synthesis of extracellular structural proteins after an injury or a pathogen attack, as well as their subsequent incorporation into cell walls *via* oxidative cross-links, has frequently been reported [25–28]. During their immobilization in the cell wall, these proteins can be linked to other extracellular compounds [29, 30] or to other proteins [31–33]. In this study, even though proteins were detected in blocked xylem vessels, extensins were not localized either in tyloses or in the amorphous material occluding xylem vessels.

#### 3.4.3. Lignins

**Figure 6:**
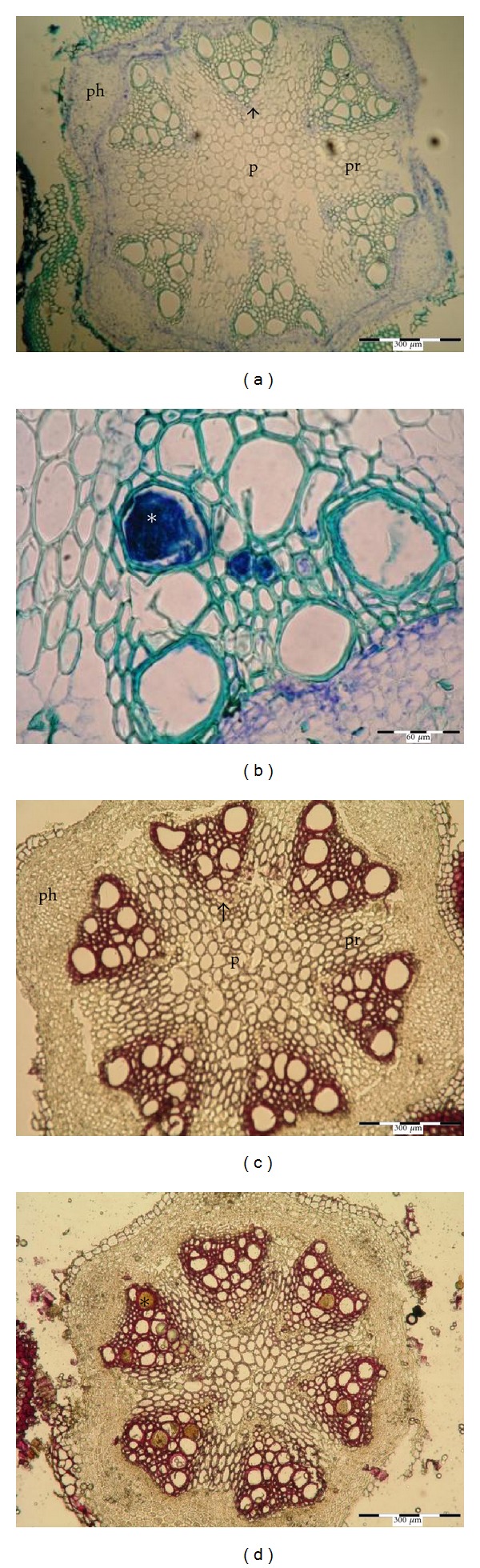
Histochemical identification of lignins in xylem occlusions in stems of *Clematis *“Solidarność” by using Azur B and phloroglucinol-HCl methods. (a) and (b) Identification of lignins in xylem of control stems; (c) and (d) Identification of lignins in xylem of stems kept in distilled water; p: pith, pr: pith rays, ph: phloem, arrow: xylem vessel, c: cortex with lamellar collenchyma, ∗: blocked xylem lumen.

Azur B showed intensive blue, and the HCL phloroglucinol showed intensive red coloration in control xylem cell walls (Figures 6(a) and 6(b)) indicating the presence of lignins. On both sampling dates, the occlusions present in stems kept in distilled water gave strong, blue coloration in metaxylem when stained with Azur B (Figure 6(c)). In phloroglucinol staining, xylem occlusions preserved their natural, orange color in contrast to strongly stained vessels (Figure 6(d)). This phenomenon appeared in xylem vessels from stems kept both in distilled water and in the preservative solution. Azur B staining of xylem blockage in stems placed in the preservative produced blue coloration of the occlusions, but the intensity was lower than in stems kept in distilled water. These confirms cytological observations that tyloses are the main cause of xylem blockage in *clematis* stems. According to Soukup and Votrubova [34] and preceding authors [15, 35], tyloses contain plenty of polyphenols which are main components of lignins and their role is mainly antibacterial. In *clematis* here, staining with Azur B was more specific for lignins relative to the HCL phloroglucinol test. The test on lignin's presence in blocked vessels clearly shows that 8HQC + SUC arrests the development of tyloses in cut stems of *clematis*, as shown by weaker color of stained occlusions. 

#### 3.4.4. Suberins

**Figure 7:**
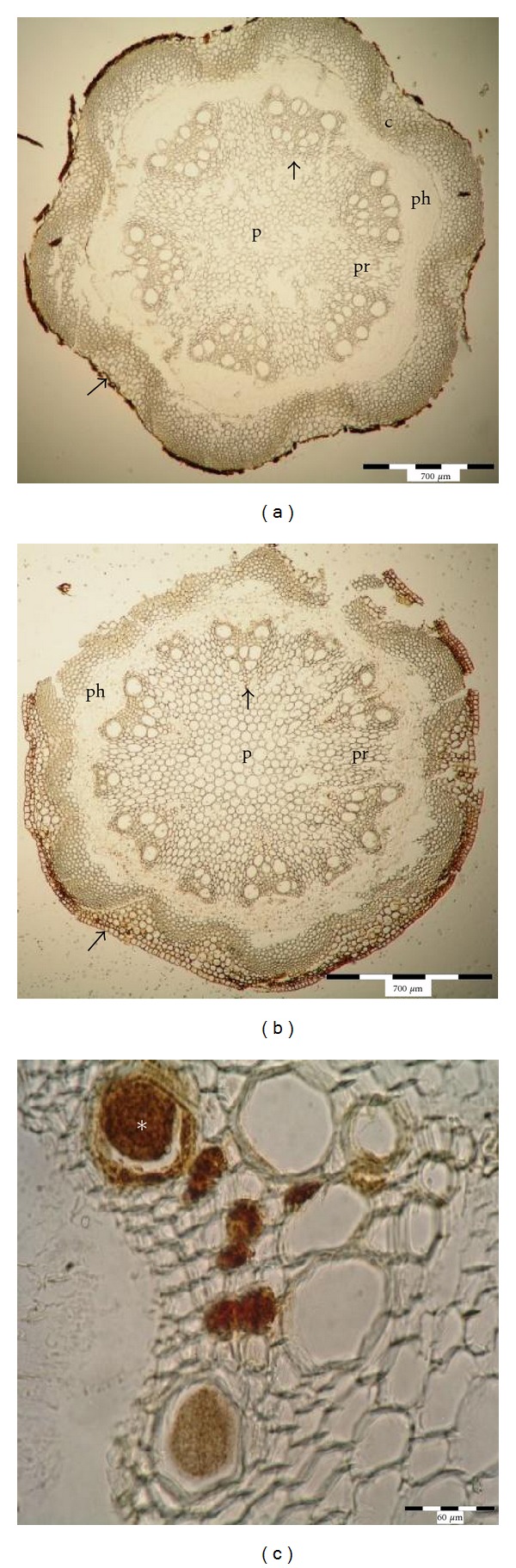
Histochemical identification of suberins in xylem occlusions in stems of *Clematis* “Solidarność” by using Sudan IV. (a) Identification of suberins in xylem of control stems; (b) identification of suberins in xylem of stems kept in 8HQC + 2% sucrose; (c) identification of suberins in stems kept in distilled water. P: pith, pr: pith rays, ph: phloem, arrow: xylem vessel, c: cortex with lamellar collenchyma, ∗: blocked xylem lumen, *↗*: epidermis.

Sections prepared from freshly harvested stems (sampling date 0) and from those kept in the solution of 8HQC + SUC, did not show any evidence of suberins (Figures 7(a) and 7(b)). Suberins were only present in xylem occlusions from stems kept in distilled water. On sampling dates I and II, suberins were present only in blocked vessels (Figure 7(c)). 

## 4. Conclusions

The main reasons of xylem blockage in cut flowers are air embolism, tyloses, plant and soil microparticles present in the water, bacteria developing in old, dirty water and spreading into vessels, and gums and gels formed in response to cutting [41, 42]. In this study, we have focused mainly on the identification of xylem occlusions appearing in the *clematis* stems during their postharvest life, depending on the keeping solution. According to Skutnik and Rabiza-Świder [1, 17], solution composed of 8HQC + SUC efficiently prolongs *clematis* vase life. 8HQC acts as a bactericide, and it may be responsible for the reduced formation of tyloses and other artifacts. According to Van Doorn et al. [43], in some woody ornamentals such as common lilac for cut flower, tylose formation is suppressed by 8HQC (8-hydroxyquinoline citrate) and AVG (aminoethoxyvinylglycine). These results were confirmed by Jedrzejuk and Zakrzewski [44] on stems of common lilac stored in distilled water, 200 mg dm^−3^ 8HQC, and Chrysal Professional. This study shows that tyloses, when they appear in *clematis* stem kept in s8HQC, they never occlude the entire lumen of a vessel as they do in stems kept in water. Tyloses produced in stems treated by 8HQC + 2% sucrose did not show the presence of degraded lipids or autophagosomal vacuoles, which is indicative of less degeneration of stem structures in comparison to stems kept in water. Histological tests revealed that the occlusions are mainly composed of proteins, lipids, polysaccharides, phenolics, and lignin-like material. These compounds were reported to appear both in woody and in herbaceous plants [24]. In *clematis*, all these components were detected in occluded vessels of the stems kept both in water and in the preservative solution. However, when stems were placed in distilled water, the percentage of blocked vessels was higher than in the stems kept in the preservative. This is demonstrated by relative differences in staining intensities in quantitative color reactions employed in this study. The 8HQC + SUC combination also significantly arrested bacterial proliferation: on the second sampling date, the percentage of vessels containing bacteria was only one half that of stems kept in distilled water (27–30%). 

This study confirms that the preservative composed of 200 mg dm^−3^ 8HQC, and 2% sucrose arrests the development of bacteria in the vessels of cut *Clematis* stems and to some extent also reduces the growth of tyloses. We also identify the origin of the xylem occlusions in *clematis* and compare their development in water and in the preservative solution. Such information can be useful to develop new treatments aiming to improve keeping qualities of cut *clematis* stems.

## Figures and Tables

**Table 1:** Histological tests used for detection of occlusions in *Clematis* stems.

Type of reaction	Color of product	Detection target
Periodic acid-Schiff reaction (PAS) [36]	Red	Aldehydes are created by oxidative cleavage of saccharides with H6IO5. Coloration is produced by the reaction
Bradford reagent [37]	Blue	Proteins
Azur B [38]	Blue	Lignins
Phloroglucinol-HCL [39]	Red	Lignins
Sudan IV [40]	Orange	Suberins

**Table 2:** Diameter of xylem lumen in 5 different cultivars of *clematis* stems.

Cultivar	Diameter of xylem lumen (*μ*m)			
	Minimum	Maximum	Mean	Standard error
Andromeda	17.9	53.9	34.8	±2.32
Viola	20.4	53.4	37.4	±7.02
Isago	25.0	66.7	44.7	±2.69
Solidarność	27.0	69.9	46.4	±2.26
Silver moon	28.9	110.5	59.0	±1.48

**Table 3:** Average number of blocked vessels (in percent) in *Clematis* stems stored in distilled water and 8HQC with 2% sucrose, term I.

Cultivar	Completely blocked vessels		Half-blocked vessels	
	Distilled water	8HQC + SUC	Distilled water	8HQC + SUC
Andromeda	7.88g	5.7e	8.64b	33.84g
Viola	5.14d	0.74b	8.72b	18.58d
Isago	7.06f	3.0c	11.0c	21.4f
Solidarność	5.66e	0.1a	9.34b	20.24e
Silver moon	7.14f	0.1a	11.36c	6.2a

Numbers followed by the same letter do not differ significantly at *P* = 0.95, according to Duncan's multiple range test, *P* ≤ 0.05.

*P*: the least significant difference.

**Table 4:** Average number of blocked vessels (in percent) in *Clematis *stems stored in distilled water and 8HQC with 2% sucrose, term II.

Cultivar	Completely blocked vessels		Half-blocked vessels	
	Distilled water	8HQC + SUC	Distilled water	8HQC + SUC
Andromeda	10.2c	28.28h	15.88b	37.54e
Viola	14.18f	11.7d	11.26a	35.48e
Isago	18.94g	11.4d	16.6b	22.36c
Solidarność	12.7e	3.74b	9.52a	28.64d
Silver moon	13.74f	2.9a	15.24b	22.6c

Numbers followed by the same letter do not differ significantly at *P* = 0.95, according to Duncan's multiple range test, *P* ≤ 0.05.

*P*: the least significant difference.

## List of Abbreviations

8HQC: 8-Hydroxyquinoline citrate
8HQC + SUC: 200 mg dm+ 2% sucrose
DEPC: Diethylpyrocarbonate
PFA: Paraformaldehyde
DMSO: Dimethylsulfoxide
NBT/BCIP: Nitroblue tetrazolium and 5-bromo-4-chloro-3′-indolyphosphate.

## References

[1] Skutnik E, Rabiza-Świder J (2005). Przydatnosc kwiatow cietych wybranych odmian powojnika (*Clematis* L.) do wykorzystania we florystyce. *Zeszyty Problemowe Postępów Nauk Rolniczych*.

[2] Van Doorn WG (1997). Water relations of cut flowers. *Horticultural Reviews*.

[3] Twumasi P, van Ieperen W, Woltering EJ, Marissen N, VanDoorn W, VanMeeteren U Effects of water stress during growth on xylem anatomy, xylem functioning and vase life in three *Zinnia elegans* cultivars.

[4] Canny MJ (1997). Tyloses and the maintenance of transpiration. *Annals of Botany*.

[5] Bretschneider KE, Gonella MP, Robeson DJ (1989). A comparative light and electron microscopical study of compatible and incompatible interactions between Xanthomonas campestris pv. campestris and cabbage (*Brassica oleracea*). *Physiological and Molecular Plant Pathology*.

[6] Boher  B, Brown I, Nicole M, Nicole M, Gianinazzi-Pearson V (1996). Histology and cytochemistry of interactions between plants and Xanthomonas. *Histology, Ultrastructure and Molecular Cytology of Plant-Microorganism Interactions*.

[7] Kpémoua K, Boher B, Nicole M, Calatayud P, Geiger JP (1996). Cytochemistry of defense responses in cassava infected by Xanthomonas campestris pv. manihotis. *Canadian Journal of Microbiology*.

[8] Rioux D, Nicole M, Simard M, Ouellette GB (1998). Immunocytochemical evidence that secretion of pectin occurs during gel (gum) and tylosis formation in trees. *Phytopathology*.

[9] Espino S, Schenk HJ (2011). Mind the bubbles: achieving stable measurements of maximum hydraulic conductivity through woody plant samples. *Journal of Experimental Botany*.

[10] Teackle DS, Smith PM, Steindl DRL (1975). Ratoon stunting disease of sugarcane: possible correlation of resistance with vascular anatomy. *Phytopathology*.

[11] Hopkins DL, Mollenhauer HH (1973). *Rickettsia*-like bacterium associated with Pierce’s disease of grapes. *Science*.

[12] Huang PY, Milholland RD, Daykin ME (1986). Structural and morphological changes associated with the Pierce’s disease bacterium in bunch and muscadine grape tissues. *Phytopathology*.

[13] Fry SM, Milholland RD (1990). Response of resistant tolerant and susceptible grapevine tissues to invasion by the Pierce’s disease bacterium *Xylella fastidiosa*. *Phytopatology*.

[14] Stevenson JF, Matthews MA, Greve LC, Labavitch JM, Rost TL (2004). Grapevine susceptibility to Pierce’s disease II: progression of anatomical symptoms. *American Journal of Enology and Viticulture*.

[15] Clérivet A, Déon V, Alami I, Lopez F, Geiger JP, Nicole M (2000). Tyloses and gels associated with cellulose accumulation in vessels are responses of plane tree seedlings (*Platanus x acerifolia*) to the vascular fungus *Ceratocystis fimbriata* f. sp platani. *Trees*.

[16] Skutnik E, Rabiza-Świder J (2006). Wplyw chlodzenia na pozbiorcza trwalosc wybranych odmian powojnika (*Clematis* L.). *Zeszyty Problemowe Postępów Nauk Rolniczych*.

[17] van Meeteren U, Arévalo-Galarza L, van Doorn WG (2006). Inhibition of water uptake after dry storage of cut flowers: role of aspired air and wound-induced processes in Chrysanthemum. *Postharvest Biology and Technology*.

[18] Beckman CH, Talboys PW, Mace ME, Bell AA, Beckman CH (1981). Anatomy of resistance. *Fungal Wilt Diseases of Plants*.

[19] VanderMolen GE, Beckman CH, Rodehorst E (1987). The ultrastructure of tylose formation in resistant banana following inoculation with *Fusarium oxysporum* f.sp. *Cubense*. *Physiological and Molecular Plant Pathology*.

[20] Bell AA, Hillocks RJ (1992). Verticillium wilt. *Cotton Diseases*.

[21] Ouellette GB, Rioux D, Blanchette A, Biggs A (1992). Anatomical and physiological aspects of resistance to Dutch elm disease. *Defense Mechanisms of Woody Plants against Fungi*.

[22] Clausen MH, Willats WGT, Knox JP (2003). Synthetic methyl hexagalacturonate hapten inhibitors of anti-homogalacturonan monoclonal antibodies LM7, JIM5 and JIM7. *Carbohydrate Research*.

[23] Niemann GJ, Baayen RP, Boon JJ (1990). Localization of phytoalexin accumulation and determination of changes in lignin and carbohydrate composition in carnation (*Dianthus caryophyllus* L.) xylem as a consequence of infection with *Fusarium oxysporum* f. sp. dianthi, by pyrolysis-mass spectrometry. *Netherlands Journal of Plant Pathology*.

[24] Rajput KS, Sanghvi GV, Koyani RD, Rao KS (2009). Anatomical changes in the stems of *Azadirachta indica* (*meliaceae*) infected by Pathogenic Fungi. *IAWA Journal*.

[25] Cassab GI, Varner JE (1988). Cell wall proteins. *Annual Review of Plant Biology*.

[26] Bradley DJ, Kjellbom P, Lamb CJ (1992). Elicitor- and wound-induced oxidative cross-linking of a proline-rich plant cell wall protein: a novel, rapid defense response. *Cell*.

[27] Tyree MT, Davis SD, Cochard H (1994). Biophysical perspectives of xylem evolution: is there a tradeoff of hydraulic efficiency for vulnerability to dysfunction?. *IAWA Journal*.

[28] Merkouropoulos G, Shirsat AH (2003). The unusual *Arabidopsis* extensin gene atExt1 is expressed throughout plant development and is induced by a variety of biotic and abiotic stresses. *Planta*.

[29] Iiyama K, Lam Thi Bach Tuyet, Stone BA (1994). Covalent cross-links in the cell wall. *Plant Physiology*.

[30] Saulnier L, Marot C, Chanliaud E, Thibault JF (1995). Cell wall polysaccharide interactions in maize bran. *Carbohydrate Polymers*.

[31] Fry SC (1982). Isodityrosine, a new cross-linking amino acid from plant cell-wall glycoprotein. *Biochemical Journal*.

[32] Biggs KJ, Fry SC (1990). Solubilization of covalently bound extensin from Capsicum cell walls. *Plant Physiology*.

[33] Brady JD, Sadler IH, Fry SC (1996). Di-isodityrosine, a novel tetrameric derivative of tyrosine in plant cell wall proteins: a new potential cross-link. *Biochemical Journal*.

[34] Soukup A, Votrubová O (2005). Wound-induced vascular occlusions in tissues of the reed *Phragmites australis*: their development and chemical nature. *New Phytologist*.

[35] Tyree MT, Zimmermann MH (2002). *Xylem Structure and the Ascent of Sap*.

[36] Pearse AG (1968). *Histochemistry (Theoretical and Applied)*.

[37] Bradford MM (1976). A rapid and sensitive method for the quantitation of microgram quantities of protein utilizing the principle of protein dye binding. *Analytical Biochemistry*.

[38] Jensen WA (1962). *Botanical Histochemistry*.

[39] Clifford MN (1974). Specificity of acidic phloroglucinol reagents. *Journal of Chromatography A*.

[40] Brundrett MC, Kendrick B, Peterson CA (1988). Efficient lipid staining in plant material with Sudan Red 7B or fluoral yellow 088 in polyethylene glycol-glycerol. *Biotechnic and Histochemistry*.

[41] Van Doorn WG, Cruz P (2000). Evidence for a wounding-induced xylem occlusion in stems of cut chrysanthemum flowers. *Postharvest Biology and Technology*.

[42] Loubaud M, Van Doorn WG (2004). Wound-induced and bacteria-induced xylem blockage in roses, *Astilbe*, and *Viburnum*. *Postharvest Biology and Technology*.

[43] Van Doorn W, Harkema H, Otma E (1991). Is vascular blockage in stems of cut lilac flowers mediated by ethylene?. *Acta Horticulturae*.

[44] Jedrzejuk A, Zakrzewski J (2009). Xylem occlusions in the stems of common lilac during postharvest life. *Acta Physiologiae Plantarum*.

